# Reconstructing Social Relationships in a Post-Lockdown Suburban Area of Southern Italy Using Pastoral Counselling

**DOI:** 10.1007/s11089-022-00999-0

**Published:** 2022-03-04

**Authors:** Antonio Stizzi, Ester Negrola, Erika Iacona, Maria Naglieri, Giorgio Scalici, Ines Testoni

**Affiliations:** 1 Theological Faculty of Puglia, Largo S. Sabino, 1, 70122 Bari, Italy; 2 Department of Philosophy, Sociology, Education and Applied Psychology (FISPPA), University of Padova, Via Venezia 14, 35131 Padova, Italy; 3 Fondazione Opera Santi Medici, Piazza Mons Aurelio Marena, 34, 70032 Bitonto (Ba), Italy; 4 Instituto de Etnomusicologia – Centro de Estudos em Música e Dança, Faculdade de Ciências Sociais E Humanas, Universidade Nova de Lisboa, Av. de Berna, 26 C, 1069-06 Lisboa, Portugal; 5 Emili Sagol Creative Arts Therapies Research Center, Faculty of Social Welfare and Health Sciences, University of Haifa, Haifa, Israel

**Keywords:** Spirituality, Pastoral counselling, Contexts of psychosocial deprivation, COVID-19 pandemic

## Abstract

The growing interest in spirituality has enabled numerous avenues of pastoral counselling support, which can be a useful resource for improving quality of life in the context of significant social deprivation. The aim of this research was to investigate the role of the spiritual dimension of pastoral support interventions created to help the inhabitants of a strongly deprived territory in Southern Italy during the COVID-19 pandemic. Eight people between the ages of 28 and 67 took part in the study. A qualitative research design was applied via online interviews with the participants, who were operators of a pastoral counselling service located on the outskirts of a suburban town. The main emergent themes were the importance of religiosity and spirituality in the lives of the participants, the role that these two aspects play in the lives of those who carry out activities devoted to helping others, and the ways in which these dimensions are used within support programmes responding to the needs of an area characterized by socioeconomic and psychosocial problems. The interviews revealed how pastoral counselling can be useful in situations of stress in highly deprived areas.

In recent years, there has been a growing interest in spirituality and religion and recognition of their role in people’s lives, but the two terms are often used interchangeably (Testoni et al., 2019b). Although both dimensions seem to have a positive impact on an individual’s well-being, they may involve different psychological traits. According to the definition of Pargament et al. (2013), spirituality refers to an individual’s ability to connect with their transcendent dimension, i.e. to experience going beyond oneself. We use the term *religion* to refer to the space in which the sacred is entrusted to language and ritual regulation through a series of practices specific to a given religious community.

Precisely because of the fundamental role of religion and spirituality in the lives of individuals, pastoral counselling is gaining interest in the field of psychology. In fact, religiosity has been seen to contribute to an increase in well-being (Rose et al., 2001; Wyatt & Johnson, 1990). In recent decades, people’s need to be understood on a spiritual level has also increased, and it is in this context that pastoral counselling is useful (Rose et al., 2001). The main goal of pastoral counselling is the convergence of theology and psychology (Pedhu, 2020), through the use of spiritual resources and psychological knowledge, for the care and growth of the person being counselled. This practice harmonizes the psychological dimensions of counselling with confessional, theological, and ethical issues (Snodgrass, 2015). To help both individuals and more complex contexts, such as families and different territorial realities, practitioners start from the life experiences of the individuals while referring to religious teachings (du Plessis, 2021). The strengths of this method are its capacity to enhance the spiritual dimension, understood as a dialectical and trusting relationship with transcendence, with others, and with oneself (Desrosiers et al., 2011; Koenig, 2015), and to guide everyday intimate and relational life (Norenzayan, 2013). Such assets are highly beneficial to community social construction because they promote trust, understanding, and forgiveness (Testoni et al., 2018a). Terror management theory has also exposed how religious and cultural defences against death anxiety influence altruistic and moral behaviour (Jonas & Fischer, 2006; Testoni & Falletti, 2016). In this area, cultural and religious variables correlate with stable relationships and self-esteem, which together constitute a safety system that helps manage the terror caused by death awareness (Hart et al., 2005).

For these reasons, we used the tool of pastoral counselling during the COVID-19 pandemic in a particularly deprived suburban area in Southern Italy. This was a working-class neighbourhood built 40 years ago with a commercial area integrated into the urban fabric. The commercial activity, however, has not been supported by sufficient investments or adequate communication networking with the rest of the provincial and regional territory, and this has led to serious and progressive impoverishment of the area. Therefore, illicit trade, mafia networks, drug use, and illegal occupation of housing have become problematic. Thus, commercial activities have abandoned the neighbourhood permanently, and the premises, left vacant, have become home to a growing population of squatters. The presence of numerous families with young children has also increased the population of school-aged children. Given their precarious socioeconomic conditions, these children show signs of increasing loss of the minimum guarantees of childhood, such as the right to grow and be educated according to the values of civil and democratic coexistence. In 2004, this area became famous due to the death of a 2-year-old girl who had been left by her family to die of hardship. This episode made this neighbourhood a symbol of the degradation of the urban suburbs of Southern Italy and illustrates how the abandonment of institutions has caused serious economic and cultural backwardness.

During the COVID-19 pandemic, religion was almost the only form of support that inhabitants of this area could count on.

## The pastoral counselling project: objectives, methods, strategies, and coordination

The objective of the pastoral counselling project, La Tela di Penelope (Penelope’s Canvas), was to activate a centre for listening and social guidance in attempt to reweave the fabric of social relations and ties to the surrounding communities following a mandatory COVID-19 lockdown period. The growing psychological and existential discomfort shown by the families in contact with the parish during the lockdown period from March 2020 to May 2020 highlighted an ever-increasing need for accompaniment. This growing need was the reason for activating the pastoral counselling service.

The human resources employed were a total of eight counsellors (two psychologists and five voluntary workers from the nonprofit organisation Caritas Italiana, founded in 1917, which is the pastoral organism of the IEC (Italian Episcopal Conference) for the promotion of charity. Its purpose is to promote charity in the Italian ecclesial community, in particular towards the most disadvantaged people, with a prevalent pedagogical function. It works in cooperation with the other 220 diocesan Caritas organizations involved in the territory and with the national ones. The objectives were to create a space for psychological and relational support in coping with the emotional and economic impacts of the COVID-19 emergency, to offer support and collaboration to the city’s welfare network, and to take charge of the concrete and spiritual needs of people. The group of counsellors began with a training course on the project led by one of the authors of this article. This training was to create a close-knit group, competent in regard to the objectives of the project and the methodology to be used, that could offer appropriate responses from a psychological and spiritual point of view. The group continued to meet weekly for interviews to ensure care and transparency in communications, thus preventing the development of ambiguous communication both within the working group and from the working group to the families that used the service.

The dialogue was centred on nonjudgmental listening but also on the reflection of concrete and spiritual suggestions. The duration of the project was two months. The pastoral counselling was conducted for two days a week at the parish premises, according to pre-established appointments. All the meetings took place in total confidentiality in a protected environment.

An average of 18 people, representatives of families, participated each day that the service was open, for a total of about 100 people throughout the 2-month study. The service was mostly used by families with children or adolescents (90%). Over the course of the 2 months, the various participants were reunited at intervals of 2 or 3 weeks, depending on the identified spiritual, socioeducational, welfare, and sometimes health needs and the presence and ages of minors. The greatest needs were for social redemption to guarantee a future for the children and for help with the additional problems caused by the pandemic.

At the end of the appointment, each person was given a food package that varied according to the number of people and minors in the household and the specific needs identified, always in proportion to the available resources. Over time, in addition to food parcels, a closet was set up in which clothes in good condition were made available to those who needed them.

## Research objectives

The study examined the perceived effectiveness of the pastoral accompaniment offered between May 2020 and July 2020 by counsellors involved in the project. The aim was to investigate how spirituality helped the counsellors’ work and to evaluate the possible positive effects on the community once the project was concluded. In addition, we wanted to concretely understand how the spiritual dimension was combined with practical help.

## Participants and methods

The group of participants consisted of the eight professionals who took part in and guided the support process. Participants ranged in age from 28 to 67 years, with a mean age of 40 years (SD = 11.71487; 50% women).

A qualitative research design was used. Participants underwent in-depth interviews via phone, Skype, and Zoom, based on their preference, with an average of approximately 60 min per call. Interviews and subsequent analyses were conducted seeking to accommodate and respect subjective opinions and viewpoints as well as possible (Camic et al., 2003; Smith, 1995; Testoni et al., 2018b, 2019a, 2021a). The researchers used an inductive, bottom-up approach which enabled them, through an actively reflexive attitude, to identify analytical categories from the transcripts of the interviews, which became explicit as the analysis proceeded. This approach allowed the emergence of unexpected themes from the texts which were not derived from predetermined hypotheses in the existing literature (Testoni et al., 2019a, 2021a). All conversations were recorded and transcribed verbatim. The resulting textual corpus was subsequently subjected to reflexive thematic analysis, which allowed the interviews to be divided into main concepts or themes (Braun & Clarke, 2006; Marshall & Rossman, 2011; Testoni et al., 2018c). After analysing the material as a whole, the work focused on an in-depth reading of the correspondence between the texts and the categories to identify central themes and any other unexpected subthemes that might have emerged as the analysis progressed (Testoni et al., 2019a; Tong et al., 2007).

The third phase was further divided into four main steps: coding the data, verifying emerging understandings, searching for alternative explanations, and writing the report (Testoni et al., 2020, 2021b). The study followed the American Psychological Association’s Ethical Principles of Psychologists and Code of Conduct and the principles of the Declaration of Helsinki. It received research ethics approval from the Ethical Committee for Psychological Research of the University of Padua (reference: 1D0278C216A4AF1B831782B58E31F3B6). Participants provided written informed consent before participating in the study.

The objectives of the research and the methodology of the analysis were explained in detail to the participants. They were asked for permission to record the conversations, transcribe their responses, and analyse their content to study the phenomenon. We promised to anonymize the contents of the obtained texts, and only those participants who gave written and signed consent participated in the research. All the names mentioned below are pseudonyms, and the quotes have been slightly modified to prevent any possibility of identification.

## Results

From the analyses, three themes emerged: “The role of spirituality and religiosity within the project”, “The needs of the territory”, and “The power of listening to construct the community”.

### First theme: the role of spirituality and religiosity within the project

In the interviews, the participants discussed the nature and importance of spirituality. According to Sergio (44 years old, a priest, who for the last two years has been in charge of the diocese in which the project was organised),

Spirituality is that irrepressible direction in every human being. That’s what I think spirituality is, which leads us to transcend our limitations. It is important to make a distinction between spirituality and religiosity. Spirituality in many cases is not expressed in a ritualization, in a collective participation, or in an identification with a specific religious belief. Spirituality is more linked to an empathetic relationship with others and with humanity.

Similarly, Marinella, 47 years old, a housewife, and a believer and practitioner who helps in the parish as a catechist and volunteer, said:

Spirituality is being in contact with the Holy Spirit. It manifests the action of God. This means that it is not faith in itself, believing and that is enough, but rather it is being moved within faith by the action of the Spirit. It is action.

All participants agreed that spirituality played a key role in the successful continuation of the journey. Sergio emphasized:

Caring for those in need is the implementation of spirituality. Action illuminated by spiritual light moves one to care for others in need. Spirituality makes one see the need of others as a mission, a calling. When this is not there, individuals ask themselves, ‘But what makes me care about others?’ The spiritual dimension elevates the gaze from the transcendent dimension.

In Marinella’s opinion, spirituality is a driving force in any mission to help others: ‘The spiritual call has united us and worked through us, moving us towards those who were asking for help. It is our driving force.’ Givanni emphasized that spirituality is a fundamental dimension of every human being, regardless of religiosity:

People who want to help others and who dedicate their time to supporting them act out of their spiritual depth, regardless of religious denomination. What really matters is that all human beings, regardless of their religiosity, can act and collaborate thanks to the spiritual drive that comes from within and is expressed by building relationships of mutuality.

Patrizia, 46 years old, graduated in psychology and is a believer and practitioner. She was hired to train the project volunteers and supervise the participants. She affirmed that spirituality is an indispensable element within support programmes because it allows one to approach others with a nonjudgmental attitude:

It is possible to help those in need only from a nonjudgmental attitude, that which is born from a deep spirituality. It is only by being led by this inner strength that it is possible to stay close to those in need without the risk of judging them or pitying them, thus not recognizing their dignity.

Participants also underlined the role of the religious dimension, considered different from spirituality. In particular, Alberto (43 years old, part of the police force), who is a believer and practitioner who does voluntary work in his parish when he can, affirmed:

Religious faith, and in particular, Christianity, are extremely important for dimensions such as charity and the choice to help one’s neighbour from a material as well as spiritual point of view. Christianity teaches what it means to be committed to the good of others. But without the pull of spirituality, religion can do nothing.

For Marinella, religiosity is the primary source of hope:

Linking one’s faith to religious content allows one to face the difficulties of life and the problems of the world trusting in God. Knowing that the Lord repays everything helps not to lose courage and confidence. We ourselves have become, as it were, the strength and hands of God in standing by, in supporting people who are in a state of need.

### Second theme: the needs of the territory

During the interview, problems related to the territory also emerged. The urban conditions of the suburbs were indicated as problematic by everyone, as Giovanni, 28 years old, a psychologist and psychotherapist in training (he does not define himself as a religious person as he is not a believer), explained in some detail:

There are structural problems, now unsustainable. It is necessary that the institutions take charge of the problem and make a radical change. The inhabitants of this neighbourhood are isolated from the rest of the province and the region. The lack of regular lines of transport and communication with the rest of the territory make it so that the neighbourhood, little by little, becomes more and more impoverished. The lack of external contacts prevents exchange and turnover and hinders all productive and commercial initiatives.

Moreover, there is a lack of places for sharing in which to plan social and political change. The parish is the only place where this happens, but there is no political and decision-making power that allows those who gravitate there to change things. Only volunteerism can work, but it is not enough. Voluntary associations that try to operate by pivoting to the parish are insufficient and cannot replace institutions and political responsibilities. As much as some citizens of good will try to change the situation, with the strength of their faith, in fact they cannot change this scenario, now deeply contaminated by illegality.

Sergio, after reiterating this same idea, underlines the following:

This condition of territorial isolation has in fact formed the neighbourhood into a ghetto. It might seem strange that in the twenty-first century it is difficult to move a few kilometres, but here this is still a problem! It is becoming increasingly difficult for families to provide a socially healthy environment for their children!

On the theme related to educational difficulties, Isabella, a 67-year-old retired woman who is a believer and practitioner, says:

Here, we constantly have to deal with what we call ‘lost children’, that is, children who don’t have someone to offer them age-appropriate social spaces, so they live on the streets. They don’t have fathers to guide them along the path of growth. They are children who organise themselves and have no point of reference. They educate themselves in street life, without adult guidance, and often drop out of school early. When here in the parish we try to create useful socialization situations to help them, these children and adolescents do not participate; they immediately abandon the initiatives because they don’t respect the adults or the rules. They already feel like adults at 8 or 9 years old. It would be necessary to open meeting centres but also educational centres to which they could be entrusted to change their destiny, which seems already determined. We would need communities to which we could entrust them in order to help them grow.

Giovanni also emphasizes the lack of cultural tools and knowledge of available services:

In this area, the educational emergency and psychosocial needs are huge. The lack of adult points of reference for these young people risks disaster. Yet, I believe that right here there is the possibility of doing a great work of social reconstruction of the community and of positive relationships. The lack of cultural resources is causing these people to dry up, and ignorance is preventing them from becoming agents of change and improvement. The parish is trying to do this, but it cannot take on such a task alone. Many people also do not access services because they do not know about the services.

### Third theme: the power of listening to construct the community

In every interview, the importance of knowing how to listen in a nonjudgmental way was emphasized. As Giulia, a 53-year-old volunteer who is a believer and practitioner, said,

People at first only came to pick up food packages, and then they realised that we were there to feed their need to be heard as well. Gradually, the need to be heard became their priority need. The nonjudgmental listening allowed them to create a bond, so the content of the packages became secondary to the possibility of talking about their condition and the need to find a solution. If I’m honest, I was a bit surprised because I didn’t expect that listening could do so much and even become the most important help. Before, I had never had the opportunity to realise how much more important it is than, for example, responding to a material need. This project also took it a step further. It allowed those who asked for help to become in turn useful for the parish and the Church.

Isabella’s narrative is also along these lines:

People eventually came just to talk to each other, to get rid of the anguish of their lives and the fear of the consequences of the pandemic, and to talk about this nightmare and their days. Sometimes they came out happy. A relationship of trust was created between us.

Patrizia emphasized how trust is the foundation of being able to build social bonds:

Yes, if there is no trust, there is also no solidarity and therefore it is impossible to build community. We need work that restores network ties. It must be a network that pivots on the authentic interests of the community by restoring a disinterested value to politics. Political corruption makes this impossible because resources are diverted to private and particular interests.

Sergio emphasizes how the bond of trust built at the parish level made it possible to find the funds to carry out the project:

This initiative of spiritual and, symbolically, also material support to the territory was made possible by the relationships of trust and solidarity that were created by sharing religious beliefs. The money collected to do all this was voluntary donations from people who attend the parish to pray and to share socially useful goals. No funds have been requested from local facilities to ensure a certain amount of freedom of action.

Giovanni emphasized the non-welfarist nature of listening:

The help offered by listening is not welfarist because it allows the needy to feel looked at and recognized, therefore, to see on their part their condition with the eyes of the listener. And this moves them to change. The person who tells you about himself opens up in dialogue, thus coming out of his own shelter, and this is already the first move towards change. It would be important to be able to continue this project to support this process of awareness raising. Unfortunately, the local counselling centre, which is supposed to do this, is closing, and, moreover, it only deals with court cases. They do not know at all what psychological and spiritual support is. The need for these people to be listened to is huge, huge.

Sergio reiterates the fact that listening has also come through symbolic help:

We did not solve material problems with our boxes of food and decent clothing. We only witnessed our presence. Our boxes symbolized our conscious listening to the needs of the area and our nonjudgmental ability to recognize them. We made it known that we were there to welcome people’s needs because the state of need is an existential condition from which we can start to build a new community together. In this way, we make people understand that they need to work to solve the problem. But we must all do it together. In this sense, the Christian faith provides relief, but the words that spiritual support brings to the table serve to make us aware of the condition in which we find ourselves and thus to act for change. This neighbourhood must realise that it must change the way it manages public affairs.

## Discussion

Spirituality and the desire to build a more just community seem to be strongly intertwined themes. In fact, according to the participants in this study, religion and spirituality were the basis of their choice to put themselves at the service of the community, giving them strength and courage even during the difficult post-lockdown period. However, as previously expressed within this research, the participants use the terms *spirituality* and *religiosity* interchangeably. As highlighted by Testoni et al. (2019b), this is a widespread phenomenon. As the literature has already pointed out in other critical situations, spirituality seems to be a complementary aspect to health care as it is a key factor in reducing the psychological effects of traumatic events and anxiety (Gonçalves et al., 2015; Hai et al., 2019). In fact, increasing levels of spirituality may potentially prove to be an important strategy to curb the effects of psychological distress (Suryani et al., 2011), post-traumatic stress disorder, and anxiety (González-Sanguino et al., 2020). Our study also shows that pastoral counselling has the potential to address the needs of particularly deprived territories. Based on the testimonies brought by the participants, it appears that the users of their service needed a listening ear and a safe place to confide in someone else and to confront their problems or even simply feel supported. As discussed in other studies, feeling heard, welcomed, and understood helps reduce stress (Hackney & Sanders, 2003; Ryan et al., 2005).

However, what we want to emphasize in particular is that the spirituality and religious beliefs of the research participants allowed them to look at the users of their services not simply as people in need of assistance but as citizens who need to become aware that their conditions derive from the lack of commitment from institutions and politicians. Looking at the value of justice is typical of believers. A systematic review by Kress and Elias (2000) describes how greater religiosity corresponds to less antisocial behaviour; therefore, adherence to religion promotes greater adherence to norms, prosociality, and the maintenance of socially approved behavioural patterns in reference to a religious context.

This mindset allowed our participants to accurately diagnose the difficulties of the context in which they operated. Thus, the project was designed to promote prosocial values that can offer a better future to children and youth who are growing up without positive adult role models. The target group that the participants addressed with their pastoral counselling support was made up of members of families who suffer from economic, social, and health difficulties of various kinds. In such a context, interventions such as the one described here can be helpful in allowing for more effective stress management and can also offer a change in direction for a community which is becoming aware of its own crisis conditions. A study by Pargament et al. (1990) has shown that a higher level of religious maturity corresponds to greater coping skills; in fact, the involvement in a religious community and the belief in a benevolent and cooperative deity offers adequate strategies for coping with the difficulties of life, increasing the values of optimism, hope, and confidence in the future (Kress & Elias, 2000). Increased coping skills and the opportunity to follow adaptive patterns would, therefore, allow people to acquire effective strategies to solve problems that arise by decreasing social degradation.

All of this, according to our participants, is related to the ability to reconstitute the solidarity of a social network through trust, which starts from spiritual experience and shared beliefs. Spirituality and religion, in this sense, are not simply individual dimensions but are also collective and social dimensions and are therefore capable of building community (Pargament, 2007).

## Conclusion

For some years now, psychology has been giving increasing importance to the dimensions of religion and spirituality in people’s lives. Today, religion and spirituality are valued for their importance not only in the construction of individual and social identity but also in the construction of the bonds of solidarity and trust on which the community is based. In fact, these dimensions have the capacity to act as protective factors in dealing with adverse events and in this way to help individuals to build a greater sense of belonging.

In conclusion, we can say that the pastoral counselling project was evaluated very positively by the participants, who made themselves available to help the population of a particularly deprived neighbourhood. Our impression is also that they were very competent in recognizing the problems of the area and its social needs.

The main limitation of this research, caused by the pandemic and also by the distance between researchers and project participants, was the fact that the interviews were conducted through an online platform. Furthermore, for privacy reasons, it was possible to interview only the members of the team and not the people who turned to this service, whose opinions could make this investigation more thorough and complete.

From the interviews, a highly interesting theme emerged several times, always related to the theme of spirituality in an area subject to criminal activities. That theme is how people belonging to a mafia organisation are connected and the roles that religion and spirituality play in their lives. This is a topic that could be explored through further research.
